# Evaluation of *Beclin1* and *mTOR* genes and p62 protein expression in breast tumor tissues of Iranian patients

**DOI:** 10.22099/mbrc.2023.47597.1837

**Published:** 2024

**Authors:** Maryam Adelipour, Mahshid Naghashpour, Mohammad Reza Roshanazadeh, Hadi Chenaneh, Asma Mohammadi, Pegah Pourangi, Seyed Rouhollah Miri, Atefeh Zahedi, Mahmood Haghighatnezhad, Sahar Golabi

**Affiliations:** 1 Department of Biochemistry, Faculty of Medicine, Ahvaz Jundishapur University of Medical Sciences, Ahvaz, Iran; 2 Department of Nutrition, School of Medicine, Abadan University of Medical Science, Abadan, Iran; 3 Department of Biochemistry, School of Medicine, Abadan University of Medical Science, Abadan, Iran; 4 Department of surgical oncology, Cancer institute, Tehran University of Medical Science; 5 Asadabad School of Medical Sciences, Asadabad, Iran; 6 Department of Physiology, School of Medicine, Abadan University of Medical Science, Abadan, Iran

**Keywords:** Autophagy, Beclin-1, Breast cancer, mTOR, Tumor grade

## Abstract

Autophagy is a cellular process that plays a major role in the fate of tumor cells. Understanding the role of autophagy in cancer therapy is a major challenge, particularly for breast cancer as the sole top cause of mortality among women. In this study, we evaluated the gene expression of *mTOR* and *Beclin1* and the levels of p62 protein, in breast tumors and compared them to a control condition. To explore the role of autophagy in breast cancer, we acquired tumor biopsies from 41 new cases of breast cancer patients. We extracted total RNA from each biopsy and used real-time PCR to quantify *Beclin1* and *mTOR*-specific RNA expression. In addition, we evaluated the expression of the p62 protein in paraffin-embedded tumor tissue using the immunohistochemistry technique. The data revealed an upregulation of *Beclin1* and a downregulation of *mTOR* in tumor tissues compared to the control condition. The correlation between p62 expression and *Beclin1*/*mTOR* showed a negative and positive correlation, respectively, confirming autophagy activation in the tumor tissues. However, there was no correlation between autophagy markers and tumor size, grade and stage. The findings revealed that autophagy activation was found in breast tumor tissues, suggesting that autophagy can be a target for breast cancer therapy.

## INTRODUCTION

Autophagy is a cellular process that degrades damaged components that helps maintain cellular homeostasis and survival by preventing toxic protein accumulation. There are three types: chaperon-mediated, microautophagy, and macroautophagy [1]. In chaperon-mediated autophagy, chaperones guide proteins to the lysosome for degradation. In microautophagy, the lysosomal membrane directly engulfs cytoplasmic cargo [2-4]. Macroautophagy forms autophagosomes enclosing cytoplasm, fuses with lysosome, releases and degrades contents [1]. Macroautophagy (referred to as “autophagy” in this article) can be selective in that specific receptor proteins can recognize specific cargos [5]. The molecular pathway of autophagy involves several proteins, such as autophagy-related proteins (Atg proteins), Beclin1, and p62 (also called SQSTM1/sequestome-1). Microtubule-associated protein light chain 3 (LC3), a homolog of yeast Atg8 in mammals, indicates the amount of autophagosome formation. During autophagosome formation, p62 contacts LC3 directly and is degraded by autophagy such that the level of p62 protein is correlated with autophagy activation, negatively [6]. The autophagy pathway is also regulated by several modulators [7]. The mechanistic target of rapamycin, also known as mTOR, majorly acts in the autophagy pathway adjustment that forms the catalytic subunit of two distinct complexes of proteins called mTOR-complex 1 (mTORC1) and mTOR-C2 [8, 9]. A previous study has shown that the activation of mTORC1 suppresses autophagy, whereas the inhibition of mTORC1 induces autophagy[10]. 

Autophagy has a complex role in cancer, with proposed tumor suppression and promotion functions. Inhibition of autophagy can lead to DNA damage, genome instability, suppression of p53, and metabolic issues, promoting tumorigenesis. However, tumors can also utilize autophagy to survive during metabolic stress and rapid growth [11-13]. 

Furthermore, the potential roles of autophagy through cancer stages remain controversial. According to evidence, in the initial stages of cancer, autophagy prevents the commencement and development of tumors, whereas it contributes to the growth and promotion of tumors in late stages[14, 15]. Since breast cancer (BC) is the prevailing feminine cancer, worldwide, understanding the molecular targets and signaling pathways, such as autophagy, could revolutionize the field of BC research [16]. BC, as a heterogeneous disease, has different biological features and is classified into different histological types. BC is divided into noninvasive and invasive forms, which may consist of different stages ranging from stage 0 to stage 4 depending upon the size and type of tumor and tumor cell penetration in breast tissues [17].

In the present work, we explored whether the gene expression of *Beclin1* and *mTOR* as autophagy markers shows any difference between normal and tumour samples. The correlation of expression of the p62 protein with the expression of *mTOR* and *Beclin* genes were also determined. Moreover, to understand the potential role of autophagy in cancer development, we assessed the relationship between autophagy markers and the stage of BC.

## MATERIALS AND METHODS

**Patients and samples: **In this study, 41 tumor samples plus equal number of adjacent normal tissues obtained from breast cancer patients of Tumor Bank of Imam Khomeini Hospital, Tehran, Iran were utilized. Normal tissues adjacent to the tumor were used as respective control. All pateints signed the constent forma before sample collection. Helsinki declaration was established as the reference of approach across the study. The present study is accepted by the Ethics Committee of Abadan University of Medical Sciences (IR.ABADANUMS.REC.1397.014).

The average age of the statistical sample was 53.4 years, ranging from 35 to 76 years. No patients had a history of radiotherapy or chemotherapy, and all of them were new cases. The demographic data of patients are shown in Table 1. Frozen tumors biopsies normal tissue samples were obtained and transferred to the laboratory. The histological grade of the tumor and clinical cancer staging was determined based on standard protocols.

**Table 1:** Demographic and tumor characteristics among patients

**Characteristic**	**Patients N=41**
**Gender** (Female/Male)	41/0
**Tumor grade**	
Low Grade (Grade I/ Low/ Well differentiated & Grade II/ intermediate/ moderately differentiated) High Grade (Grade III/ High/ poor differentiated)	22 (53.6%) 19 (46.4%)
**Tumor stage**	
High Stage (IIA &IIB) Low Stage (IIIA & IIIB)	24 (58.5.9%) 17 (41.5%)
**Tumor Size (cm)**	
<5 ≥5	32 (78%) 8 (22%)
**ER Status**	
Positive Negative Missing	8 (19.5%) 14 (34.1%) 21 (51.2%)
**PR Status**	
Positive Negative Missing	13 (31.7%) 13 (31.7%) 15 (36.5%)
**HER2 Status**	
Positive Negative Missing	17 (41.4%) 9 (21.9%) 15 (36.5%)
**P53 Status**	
Positive Negative Missing	11 (26.8%) 9 (21.9%) 21 (51.2%)

**RNA extraction and cDNA reverse transcription: **Tissue total RNA isolation was conducted by the Hybrid-R RNA isolation kit (Gene All, Korea), as per the producer's protocol. The product concentration was determined by NanoDrop 2000C spectrophotometer (Thermo Scientific, Wilmington, DE, USA). Furthermore, the integrity of RNA isolate was determined by agarose gel electrophoresis. Then, cDNA was synthesized using the HyperScript RT master mix (Gene All, Korea) in accordance with the manufacturer’s protocols.

***Beclin1*** **and** ***mTOR*** **gene expression:** The levels of *Beclin1* and *mTOR* genes expression was quantified through qRT-PCR by a master mix of SYBR green (Ampliqon, Denmark) in the LightCycler 96 Real-Time PCR instrument (Roche, USA). The primer pairs for the genes were as follows: *mTOR*: Forward, 5’CCAAAGGCAACAAGCGATCC-3’, Reverse, 5’-TGAGAGA AGTCCCGACCAGT-3’; *Beclin1*: Forward, 5’-GGAGCTGGAAGACGTGGAAAA-3’, Reverse, 5’-AGGTTGCATTAAAGACGTTGG-3’. At the same time, the hypoxanthine phosphoribosyl transferase 1 (HPRT1) gene was selected as our internal reference with the following primer pair: Forward, 5’-CCTGGCGTCGTGATTAGTG-3’, Reverse, 5’- TCAGTCCTGTCCATAATTAGTCC-3’. The following PCR cycles were applied: 95ºC for 15 min, 15 s at 95°C and 1 min at 60ºC for 40 cycles. 

**p62 expression by Immunohistochemistry: **In this study, p62 expression in tumor tissues was estimated using an anti-p62 antibody. First, 4 µM sections were prepared from paraffin-embedded tumor samples, stained with an anti-p62 primary antibody for 45 minutes, rinsed by PBS, maintained with secondary antibody with HRP-labeling, and then rinsed and developed with diaminobenzidine tetrahydrochloride (DAB). These steps were taken according to the routine protocols of the cancer institute, Imam Khomeini Hospital, Tehran, Iran. The immunoreactive cells were counted under a light microscope using a 40x lens (400x magnification) and reported as p62 positive or negative [18, 19].

**Statistical analysis:** The analysis of the acquired data was conducted using SPSS 26.0. The data normality was determined utilizing Kolmogorov-Smirnov test. Through Mann-Witeny test, gene expressions were compared between the two groups. A comparison between gene or protein expression and clinicopathologic parameters was also performed using a Mann witeny-test. Correlation of between autophagy markers expression was evaluated by the spearman test. The statistical significance level was considered at p<0.05.

## RESULTS

**Figure 1:**
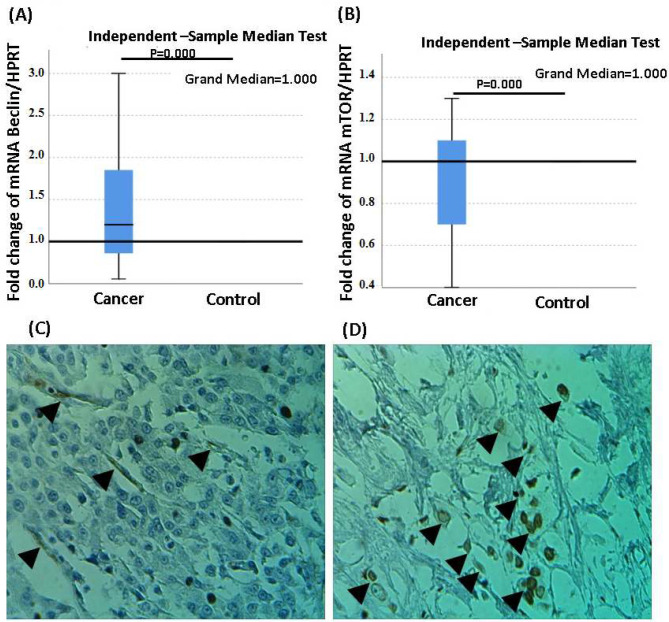
*Beclin1* and *mTOR* gene expression and p62 protein expression in tumor tissues.

Through Mann-Whitney test the median scores of cancer tissues (n=41) and non-cancer adjacent tissues (n=41) on the *Beclin1* mRNA expression were compared. The test statistic was U=30.06, p<0.001, which presents a significant difference between the two groups (Fig. 1A). Furthermore, the *mTOR* expression significantly decreased in tumor tissues compared to controls (U= 23.06, p<0.001) (Fig. 1B). The expression of p62 protein in breast tumor tissue was evaluated using immunohistochemistry and classified as high-expressed p62-tumors or low-expressed p62-tumors, as shown in Figure 1 C and D. Through the correlation test of Pearson, it was shown that there is a positive correlation between the gene expression of *Beclin1* and the protein levels of p62 in the cancerous samples (R=0.773, P<0.001), which is statistically significant. In addition, a negative correlation between *mTOR* gene expression and p62 protein levels in tumor tissues (R=-0.758, p<0.001) were observed. The values -0.719 and p<0.001 are the correlation coefficients between *mTOR* and *Beclin1* gene expression (Table 2).

**Table 2:** Correlation of *Beclin1* and *mTOR* gene expression (ΔCt) and p62 protein expression

**Correlation Coefficient**	***Beclin1***	**P**	***mTOR***	**P**	**p62**	**P**
***Beclin1***	1	.	-.719**	0.000	.773**	.000
***mTOR***	-.719**	.000	1	.	-.758**	.000
**P62**	.773**	.000	-.758**	0.000	1	.

** Correlation is significant at the 0.01 level (2-tailed).

**Figure 2:**
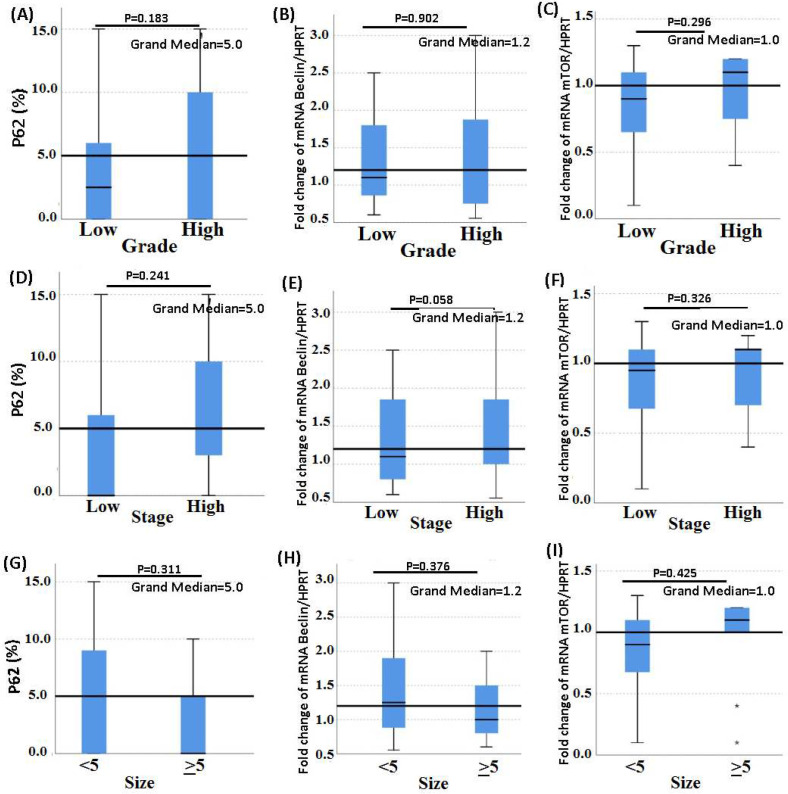
Correlation between Beclin1 and *mTOR* gene expression and p62 protein expression in tumor tissues and tumor size, grade, and stage.

To explore the fluctuations of *Beclin1* and *mTOR* genes, and p62 markers across different clinicopathological parameters, the size of the tumor was classified as <5 cm or ≥5 cm, the grade was classified as low grade (including Grade I/low/well-differentiated & Grade II/intermediate/moderately differentiated) or high grade (including Grade III/high/poorly differentiated), and the stage was classified as low stage (IIA & IIB) or high stage (IIIA & IIIB). Considering that the data were not normally distributed, through Mann-Whitney test we evaluated the difference in expression of autophagy markers. As shown in Figure 2, between the expression of *Beclin1* and tumor size (U=1.025, p=0.311), grade (U=0.015, p=0.902), and stage (U=0.006, p=0.938) no significant correlation was witnessed. Similarly, the Mann-Whitney test showed no significant correlation occurs between *mTOR* expression and tumor size (U=0.636, p=0.425), grade (U=1.096, p=0.295), and stage (U=0.963, p=0.326). Furthermore, the relationship between p62 and the tumor size (U=0.785, p=0.378), grade (U=1.77, p=0.183), and stage (U=1.373, p=0.241) were not significantly correlational.

(A): The *Beclin1* expression at mRNA levels by qPCR is increased in breast tumor tissue in compression with respective control. (B): The *mTOR* expression at mRNA levels by qPCR is deceased in breast tumor tissue in compression with respective control. (C): Low expression of p62 protein in tumor tissue by immunohistochemistry technique. (D): High expression of p62 protein in tumor tissue by immunohistochemistry technique.

In order to analyze the expression difference between autophagy markers and tumor size, grade, and stage, no data was missed. However, some data on the expression of p53, Her2, estrogen receptor and progesterone receptor was missing. To investigate the differences in the expression of *Beclin1*, *mTOR*, and p62 markers across ER, PR, Her2, and P53 features, two groups were described for each parameter, based on whether they were positive or negative. As presented in Table 3, the gene expression of *Beclin1* was significantly overexpressed in PR-positive breast tumors (U=48, P=0.03). Additionally, a significant rise was observed in Beclin1 expression in p53-negative tumors (U=22.5, P=0.03). Nevertheless, no significant differences witnessed between autophagy marker expression and other clinicopathological parameters.

## DISCUSSION

Autophagy, as an intracellular catabolic pathway, is effective in the fate of tumor cells [20-23]. Since, in BC, autophagy acts paradoxically, in this study, the expression of *Beclin1* and *mTOR* genes, as autophagy markers, were evaluated in breast tumor biopsies compared to respective controls. According to our data, there is a significant upregulation of *Beclin1* as well as a significant down-regulation of *mTOR* at mRNA levels in tissue biopsies of breast cancer which can indicate the activation of the autophagy pathway in breast tumor tissues (Fig. 1A and B). Regarding the degradation of p62 protein during autophagy flux, the expression of this protein was shown by the immunohistochemistry technique. 

Graphs A, B, and C show the differences in *Beclin1* and *mTOR* gene expression and p62 protein expression between low- and high-grade breast tumors. Graphs D, E, and F show the differences in *Beclin1* and *mTOR* gene expression and p62 protein expression between low- and high-stage patients. Graphs G, H, and I show the differences in *Beclin1* and *mTOR* gene expression and p62 protein expression between tumors with sizes <5 and ≥5.

**Table 3:** Correlation of *Beclin1* and *mTOR* gene expression and p62 protein expression with clinicopathologic parameters in patients with breast cancer

	***Beclin1*** **Median (N)**	**U**	**P**	***mTOR*** **Median (N)**	**U**	**P**	**P62** **Median (N)**	**U**	**P**
**ER Positive** **ER Negative**	1 (8) 1.1 (14)	52	0.8	1.1 (8) 0.84 (14)	27.5	0.05	6 (8) 2.5 (14)	44	0.4
**PR Positive** **PR Negative**	1.7 (13) 0.9 (13)	48	0.03*	1.1 (13) 1 (13)	71	0.5	7 (13) 0 (13)	53	0.1
**Her2 Positive** **Her2 Negative**	1 (17) 0.8 (9)	63	0.49	1 (17) 1.1 (9)	71.5	0.79	5 (17) 5 (9)	76.50	>0.999
**P53 Positive** **P53 Negative**	0.8 (9) 1.8 (11)	22.5	0.03*	0.8 (9) 1.1 (11)	30.5	0.21	0 (9) 5 (11)	30.50	0.4

*Indicate significant difference between two statuses with P<0.05

In this study, we only had access to paraffin-embedded cancer tissue blocks and were unable to obtain normal tissue samples for comparison, which is a limitation. Therefore, p62 protein expression in tumor tissue was compared with the ΔCt value of *Beclin1* or *mTOR* gene expression in tumor tissues. Since a lower ΔCt value indicates higher gene expression, a positive correlation between the ΔCt value of each gene and p62 protein(%) indicates that lower gene expression coincides with higher p62 protein or higher gene expression coincides with lower p62 protein. Similarly, a negative correlation between the ΔCt value of each gene and p62 protein (%) indicates that higher gene expression coincides with lower p62 protein or lower gene expression coincides with higher p62 protein. Therefore, as shown in Table 2, the Pearson correlation test indicated a significant correlation positively occuring between *Beclin1* gene and p62 protein levels in tumor samples, confirming activation of the autophagy pathway in breast tumor tissue. Additionally, a negative correlation between *mTOR* gene and p62 protein levels in tumor tissues is observable, indicating inhibition of autophagy in high levels of *mTOR* expression. The correlation coefficient between *mTOR* and *Beclin1* gene expression was -0.719, P=0.000, proving the inhibitory impacts of *mTOR* on the autophagy pathways induction.

Autophagy activation in tumor samples might be one of the defense mechanisms of the cell against the shortages of oxygen and nutrients due to rapid tumor growth. Some evidence show the autophagy's influence on tumorigenesis and the survival of tumor cells. For example, Hu et al. showed that autophagy is a hypoxia-induced mechanism that enhance the survivability of tumor cells as a way to cope with anti-angiogenic administrations in glioblastoma [24]. In addition, Vera-Ramirez's study found that inhibiting autophagy in dormant breast cancer cells reduces survival and metastasis and leads to impaired mitochondria aggregation, reactive oxygen species (ROS) increase, and cell apoptosis [25]. 

According to this study, there was no significant difference between the autophagy markers *Beclin1*, *mTOR*, and p62 and tumor size, grade, or cancer stage (Fig. 2). However, due to limitations in samples number we only possessed two experimental group for tumor size, tumor grade, and cancer stage, which could affect the results of the correlation between autophagy markers and the clinicopathological characteristics of patients. 

Autophagy can also suppress oxidative stress and p53 tumor suppressor protein, promoting the survival of mammalian cells. Yung et al. indicated that the knockdown of p53 and/or Atg7 as an essential autophagy gene in mice leads to an extended life span, which occurs resulting of neuro-degeneration deferral and death resistance due to fasting. They suggested that autophagy inhibits the activation of p53 and p53-related neuro-degeneration [26]. In our study, we observed overexpression of Beclin1, an autophagy marker, in p53-negative breast tumor biopsies (Table 3). This finding is consistent with the study conducted by Yung et al. 

Regarding the correlation between autophagy and breast cancer subtype, Chen et al. found that autophagy promotes triple-negative breast cancer (TNBC) metastasis via the Hippo signaling pathway's yes-associated protein (YAP). Inhibiting YAP translocation delayed TNBC cell migration and invasion but had no effect on ER-positive breast cancer cells [27]. Our study indicated no significant differences between autophagy markers and ER or the Her2 status of breast cancer, although PR-positive tumors showed an increased expression of the Beclin1 gene (Table 3).

Two main limitations in our study were the unavailability of adjacent paraffin-embedded-normal tissues for p62 assessment and the lack of normal breast tissues as a control group. We observed significant differences in the expression of *mTOR* and *Beclin* genes between cancer and normal groups, indicating the possibility of autophagy activation in cancerous samples. Higher expression of *Beclin1*, contemporary with lower levels of *mTOR* gene and p62 protein lead to autophagy activation in tumor tissues. However, we did not find a correlation between the activation of autophagy and tumor size, grade, or cancer stage. 

### Acknowledgment:

The present study was financially supported by the Abadan University of Medical Sciences (Grant no: 96U-346).

### Conflict of Interest:

The authors declare that they have no competing interest.

### Authors’ Contribution:

HC, MR, AM and PP contributed to methodology, MA was responsible for the conduct of this study, funding acquisition, writing, review & editing, MN and SG contribiuted to the methodology, and investigation, and AZ contributed to data analysis. SM and MH contributed to sample collection. All authors read and approved the manuscript.
